# A Viral Ubiquitin Ligase Has Substrate Preferential SUMO Targeted Ubiquitin Ligase Activity that Counteracts Intrinsic Antiviral Defence

**DOI:** 10.1371/journal.ppat.1002245

**Published:** 2011-09-15

**Authors:** Chris Boutell, Delphine Cuchet-Lourenço, Emilia Vanni, Anne Orr, Mandy Glass, Steven McFarlane, Roger D. Everett

**Affiliations:** MRC-University of Glasgow Centre for Virus Research (CVR), Glasgow, Scotland, United Kingdom; McMaster University, Canada

## Abstract

Intrinsic antiviral resistance represents the first line of intracellular defence against virus infection. During herpes simplex virus type-1 (HSV-1) infection this response can lead to the repression of viral gene expression but is counteracted by the viral ubiquitin ligase ICP0. Here we address the mechanisms by which ICP0 overcomes this antiviral response. We report that ICP0 induces the widespread proteasome-dependent degradation of SUMO-conjugated proteins during infection and has properties related to those of cellular SUMO-targeted ubiquitin ligases (STUbLs). Mutation of putative SUMO interaction motifs within ICP0 not only affects its ability to degrade SUMO conjugates, but also its capacity to stimulate HSV-1 lytic infection and reactivation from quiescence. We demonstrate that in the absence of this viral countermeasure the SUMO conjugation pathway plays an important role in mediating intrinsic antiviral resistance and the repression of HSV-1 infection. Using PML as a model substrate, we found that whilst ICP0 preferentially targets SUMO-modified isoforms of PML for degradation, it also induces the degradation of PML isoform I in a SUMO modification-independent manner. PML was degraded by ICP0 more rapidly than the bulk of SUMO-modified proteins in general, implying that the identity of a SUMO-modified protein, as well as the presence of SUMO modification, is involved in ICP0 targeting. We conclude that ICP0 has dual targeting mechanisms involving both SUMO- and substrate-dependent targeting specificities in order to counteract intrinsic antiviral resistance to HSV-1 infection.

## Author Summary

Viruses must evade several antiviral defences in order to establish a productive infection. These include antibody- and cell-mediated acquired immunity and interferon-regulated innate immunity. Recently, a third arm of antiviral defence has been discovered, so called intrinsic immunity. This aspect of antiviral resistance represents the first line of intracellular defence against virus infection and is mediated by pre-existing cellular factors that attempt to repress viral replication during the initial stages of infection. Like acquired and innate immunity, viruses have evolved mechanisms that overcome intrinsic defence. Here we show that in response to herpes simplex virus type-1 (HSV-1) infection an important aspect of intrinsic immunity is regulated by the small ubiquitin-like modifier (SUMO) conjugation pathway. In response to this defence, the virus induces rapid degradation of specific SUMO-conjugated proteins, followed by widespread loss of SUMO-conjugated species in general. Inactivation of the SUMO pathway inhibits the cell’s ability to efficiently repress viral replication in the absence of this viral countermeasure. Our data identifies an important regulatory pathway that mediates intrinsic resistance to HSV-1 infection and describes the biochemical mechanism that the virus utilizes in order to counteract this antiviral defence.

## Introduction

The ubiquitin pathway regulates many essential cellular processes including protein degradation, the cell cycle, transcription and DNA repair. It is not surprising that many viruses have therefore evolved strategies to take advantage of this pathway in order to enhance their replication (for a recent review see [1]). During herpes simplex virus type-1 (HSV-1) infection, one of the first viral proteins to be expressed is ICP0 (infected cell protein 0), an E3 ubiquitin ligase of the RING finger class that is required for the efficient initiation of lytic infection and productive reactivation of viral genomes from latency (reviewed in [2]). Whilst the exact mechanisms by which ICP0 stimulates infection remain to be elucidated, it is clear that the ubiquitin ligase activity of ICP0 plays a fundamental role in regulating infection. Deletion or point mutations within the RING finger of ICP0 that inactivate its ubiquitin conjugation activity completely impair its ability to stimulate lytic infection and the reactivation of quiescent viral genomes [3]–[8].

During infection, ICP0 localizes to promyelocytic leukemia (PML) nuclear bodies (PML-NBs, also known as ND10 or PODs) where it induces the proteasome-dependent degradation of PML, its small ubiquitin-like modifier (SUMO)-modified isoforms, and SUMO-modified Sp100 [9]–[11]. Recent findings suggest that these ND10 proteins play a role in contributing to intrinsic antiviral defence, as depletion of these proteins increases the likelihood of an ICP0-null mutant virus entering productive infection [12], [13]. The available evidence is consistent with the hypothesis that ICP0 targets specific cellular proteins for proteasome-dependent degradation in order to inhibit (or relieve in the case of latency) cellular mechanisms that would otherwise repress viral transcription [14]. However, the mechanism(s) by which ICP0 targets these cellular proteins for degradation remains unclear.

One of the earliest detectable events during HSV-1 infection is a cellular response that leads to the accumulation of ND10 components at sites closely associated with viral genomes soon after they have entered the nucleus. This response is rapidly counteracted by the ubiquitin ligase activity of ICP0, a phenotype that correlates well with its ability to stimulate lytic infection and reactivation from quiescence [8], [15], [16]. We have recently shown that this recruitment is dependent upon SUMO Interaction Motifs (SIMs) within these proteins and that mutation of these motifs inhibits their abilities to repress viral replication [17]. These data implicate a role for the SUMO conjugation pathway in mediating intrinsic resistance to HSV-1 infection. However, several important questions remain outstanding. For example, how does ICP0 target PML and its SUMO-modified isoforms for degradation, what other cellular factors are also targets for ICP0-mediated degradation, and how does ICP0 inhibit the recruitment of proteins other than PML to these repressive foci associated with incoming HSV-1 genomes?

Recent findings have highlighted a link between ubiquitin ligase targeting and the proteasome-dependent turnover of SUMO-modified proteins through the discovery of SUMO Targeted Ubiquitin Ligases (STUbLs). These proteins represent a class of RING finger ubiquitin ligases that contain SIMs that were initially identified in yeast through the characterization of Slx5 and Slx8 in *S. cerevisiae*, Rfp1 and Rfp2 in *Schizosaccharomyces pombe*, and more recently RNF4 in mammalian cells [18]–[23]. These SIM-containing ubiquitin ligases provide a means of regulating SUMO-modified substrates via their ubiquitination and proteasome-dependent degradation. SIMs typically consist of a short core of hydrophobic amino acids, (V/I/L)-x-(V/I/L)-(V/I/L) or (V/I/L)-(V/I/L)-x-(V/I/L), which form a β-strand that binds in a groove formed between the α-helix and a β-strand of SUMO [24]–[26]. SIMs are often followed by acidic or phospho-serine residues that enhance the SIM-SUMO interaction [27], [28]. The best characterized mammalian STUbL is RNF4, a SIM-containing RING finger ubiquitin ligase that promotes the degradation of SUMO-modified PML following arsenic trioxide treatment. Multiple SIMs within the N-terminus of RNF4 mediate its association with poly-SUMO chains anchored on PML, leading to the ubiquitination of both SUMO and PML [22], [23], [29], [30].

Given the parallels between the STUbL activity of RNF4 and ICP0’s ability to degrade PML, and as ICP0 had previously been shown to induce the loss of SUMO conjugates following ectopic expression of tagged SUMO-1 [9], we decided to investigate if ICP0 represented a viral STUbL. Here we report that ICP0 contains multiple SIM-like sequences (SLSs), one of which shares homology to a SIM previously characterized within hDaxx and HCMV IE2, and that mutation of specific SLSs inhibits its ability to interact with and promote the ubiquitination of SUMO-2 chains. We demonstrate that during infection ICP0 induces the global degradation of high molecular weight (MW) SUMO conjugates in a RING finger- and proteasome-dependent manner, although certain SUMO-conjugates are degraded more rapidly than others. Utilizing a panel of ICP0-expressing cell lines, we show that combined mutation of several SLSs has a clear detrimental effect upon ICP0 function and its ability to degrade SUMO-conjugates. Using PML as a model substrate, we demonstrate that ICP0 preferentially induces the degradation of all SUMO-modified PML isoforms, but can additionally target PML isoform I for degradation in a SUMO modification-independent manner. We also demonstrate that the SUMO conjugation pathway plays an important role in mediating the recruitment of ND10 components to sites associated with incoming HSV-1 genomes. Depletion of Ubc9, the sole E2 SUMO conjugating enzyme, partially relieves this cell-mediated repression mechanism and increases the replication efficiency of an ICP0-null mutant virus. Taken together our data demonstrate that the SUMO conjugation pathway contributes to intrinsic antiviral resistance and that these activities are counteracted by both SUMO- and substrate-dependent targeting specificities of ICP0.

## Results

### The SUMO conjugation pathway plays a role in intrinsic resistance to HSV-1

Previous studies have shown that several ND10 proteins, namely PML, Sp100, hDaxx and ATRX, contribute to intrinsic resistance to HSV-1 infection, and that this cellular response is counteracted by the viral ubiquitin ligase ICP0 [12], [13], [31]. An important aspect of intrinsic antiviral resistance is the ability of these constitutively expressed proteins to relocate to sites associated with incoming viral genomes in order to mediate the transcriptional repression of viral gene expression [15], [16], [31]. We have recently shown that mutation of SIMs within PML and hDaxx inhibited the ability of these proteins to localize to HSV-1 genomes and thereby restrict the replication of an ICP0-null mutant virus [17]. Furthermore, in cells depleted of PML additional SUMO-2/3 conjugates were observed to localize to sites associated with incoming viral genomes [17]. These data imply a potential mechanistic role for SUMO conjugation in mediating intrinsic antiviral resistance.

**Figure 1:**
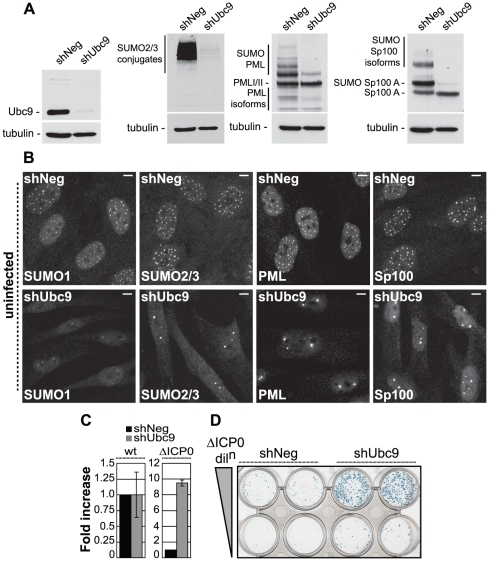
The SUMO pathway contributes to intrinsic antiviral resistance to HSV-1 infection. (A) Western blots analyzing the expression of Ubc9, SUMO-2/3 conjugates, PML and Sp100 in HFs stably expressing control (shNeg) or Ubc9 (shUbc9) shRNAs. (B) Nuclear distribution of endogenous SUMO-1 and SUMO-2/3 conjugates, PML and Sp100 at ND10 in uninfected shNeg and shUbc9 cells. Scale bar represents 5 µm. (C) shNeg or shUbc9 cells were infected with wt or ICP0-null mutant (ΔICP0) viruses expressing a β-galactosidase reporter gene from the *tk* locus. 24 hours post-infection the cells were stained for β-galactosidase expression and relative plaque formation efficiencies calculated and expressed as fold increase with respect to the appropriate control infection. Means and standard deviations of three independent experiments are presented. (D) Duplicate wells of shNeg and shUbc9 cells infected with two input doses of ΔICP0 virus and stained for β-galactosidase expression 24 hours post-infection.

To test this hypothesis, human diploid fibroblasts (HFs) were transduced with lentiviruses expressing control (shNeg) or anti-Ubc9 shRNAs and analyzed in assays monitoring intrinsic resistance to HSV-1 infection, either in the presence or absence of ICP0 (Figures 1 and S1). Ubc9 is the sole E2 SUMO conjugating enzyme and is essential for SUMO conjugation. Cells stably expressing an shRNA to Ubc9 exhibited significant depletion in Ubc9 expression, as well as decreased abundance of SUMO-1 and SUMO-2/3 conjugates in comparison to control cells (Figure 1A and data not shown). Ubc9-depleted cells also showed significant reductions in SUMO modification of both PML and Sp100 (Figure 1A), dispersal of intra-nuclear SUMO-1 and SUMO-2/3 conjugates, and dramatic changes in the number and distribution of ND10 (Figure 1B, bottom panels). These data demonstrate that depletion of Ubc9 efficiently restricts the SUMO conjugation pathway, affecting the overall abundance of SUMO-conjugates and ND10 integrity.

To investigate the role of SUMO conjugation in intrinsic antiviral resistance we examined the relative plaque formation efficiencies (PFE) of both wild type (wt) and ICP0-null mutant (ΔICP0) HSV-1 in control and Ubc9-depleted cells. Wt HSV-1 PFE was unaffected by depletion of Ubc9 (Figure 1C). In contrast, ICP0-null mutant (ΔICP0) HSV-1 infection exhibited a 10-fold increase in PFE in Ubc9-depleted cells compared to control cells (Figures 1C and 1D). Although significant depletion of Ubc9 was achieved (Figure 1A), the SUMO pathway is required for cell division [32]-[34]. In accordance, we noted that Ubc9 depletion could not be maintained long-term and that depleted cells had to be analyzed soon after isolation. Therefore, it is likely that some Ubc9 remains in order to allow limited cell division to occur while under selection. The observed increase in ICP0-null mutant PFE may therefore represent an underestimate in the repressive role that SUMO conjugation plays during HSV-1 infection in the absence of ICP0 expression. We note that depletion of Ubc9 itself does not impact upon the infection process *per se*, as wt virus was unaffected in plaque forming efficiency in comparison to control cells (Figure 1C).

We next investigated the recruitment of SUMO and PML to sites associated with incoming viral genomes in the absence of ICP0 expression (Figure S1). Viral genomes can be visualized by the appearance of punctate foci containing the major HSV-1 transcription activator ICP4 [15], which binds strongly to viral DNA. Infection of control cells resulted in a significant localization of SUMO-1 and SUMO-2/3 conjugates, as well as PML, to sites closely associated with viral genomes (Figure S1, shNeg panels). In contrast, recruitment of PML and SUMO-conjugates was greatly reduced in cells depleted of Ubc9 (Figure S1, shUbc9 panels). We conclude that the SUMO conjugation pathway is required for the efficient recruitment of intrinsic antiviral factors to sites associated with incoming HSV-1 genomes and the efficient repression of ICP0-null mutant replication.

### ICP0 induces the proteasome-dependent degradation of SUMO-conjugates during infection

**Figure 2:**
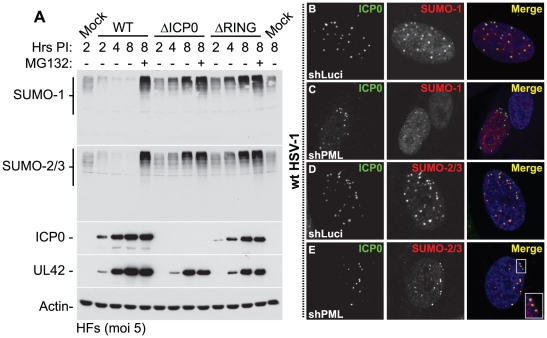
ICP0 localizes to and induces the degradation of SUMO-1 and SUMO-2/3 conjugates during infection in a RING finger- and proteasome-dependent manner. (A) HFs were infected at a MOI of 5 plaque forming units (pfu) per cell with wt HSV-1, ICP0-null (ΔICP0) or ICP0 RING finger deletion (ΔRING) mutant viruses in the absence or presence (-/+) of the proteasome inhibitor MG132. Cells were harvested at the indicated time points post-infection (Hrs PI) and analyzed for SUMO-1 and SUMO-2/3 abundance. The blots were reprobed for viral antigens ICP0 and UL42, and actin as a loading control. Longer exposures of western blots from duplicate experiments are shown in Figure S2B. (B–E) ICP0 localizes to SUMO-2/3, but not SUMO-1, conjugates in a PML-independent manner. Control (shLuci) or PML-depleted (shPML) HFs were infected with wt HSV-1 and the localization of ICP0 (green) to endogenous SUMO-1 and SUMO-2/3 conjugates (red) was analyzed in cells at the periphery of developing plaques (PML localization within these cells is shown in Figure S4). Nuclei were stained with DAPI. The insert at the lower right corner shows an expanded region highlighted by the white box.

ICP0 has been reported to induce the proteasome-dependent degradation of PML and its SUMO-modified isoforms [9]-[11], as well as other SUMO-1 conjugated proteins following the ectopic expression of myc-tagged SUMO-1 [9], [35], [36]. We therefore decided to investigate the effect of ICP0 on the stability of endogenous SUMO-1 and SUMO-2/3 conjugated proteins. HSV-1 infection of HFs at a high multiplicity of infection (MOI) of 5 plaque forming units (pfu) per cell induced a general loss of high MW SUMO-conjugates in an ICP0-, RING finger- and proteasome-dependent manner (Figure 2A, an independent experiment is shown in Figure S2B). Intriguingly, instead of a decrease, a significant increase in the levels of both SUMO-1 and SUMO-2/3 conjugates was detected at this MOI with ICP0-null (ΔICP0) and ICP0 RING finger deletion (ΔRING) mutant viruses (Figure 2A). The relative infection efficiencies of the wt and mutant viruses were compared by detection of the viral DNA polymerase accessory factor UL42. While the mutant viruses exhibited some defect in viral gene expression at this MOI, this was not sufficient to explain the differences in SUMO-conjugate expression levels during infection (Figure 2A). The C-terminus of ICP0, encompassing its USP7 binding domain and ND10 localization sequences, was also required for efficient SUMO-conjugate degradation (Figure S2C). This activity occurred in a number of other cell types, including primary keratinocytes (HaCat, data not shown) and HepaRG hepatocytes (Figure S3A). As observed previously [19], [37], addition of the proteasome inhibitor MG132 not only inhibited ICP0-mediated degradation of SUMO-conjugates, but also led to a substantial increase in their abundance (Figure 2A).

Degradation of SUMO-conjugates in general occurred less rapidly than that of PML and its SUMO-modified isoforms in both HFs and HepaRG cell types (Figure S3A). This suggests that the identity of a given substrate, as well as the fact that it is conjugated to SUMO species, influences the efficiency of ICP0-mediated degradation. These data are consistent with the initial localization of ICP0 to SUMO-conjugates localized at ND10 prior to bringing about their degradation (Figures 2B and 2D, with further details in Figures S4A and S4C). Interestingly, even in PML-depleted cells, ICP0 colocalized with SUMO-2/3 conjugates, but not SUMO-1 conjugates, at the earliest stages of infection when only very low levels of ICP0 were present (Figures 2C, 2E, S4B and S4D). Therefore, ICP0 localizes to sites that contain condensed SUMO-conjugates, either in the presence or absence of PML. ICP0-mediated degradation of SUMO-conjugates was also independent of PML expression (Figure S3B). We conclude that ICP0 exhibits properties related to those of a STUbL, inducing the rapid loss of SUMO-modified PML followed by widespread proteasome-dependent degradation of SUMO-conjugates during infection in a RING finger-dependent manner.

### ICP0 interacts with SUMO in a SIM-dependent manner

**Figure 3:**
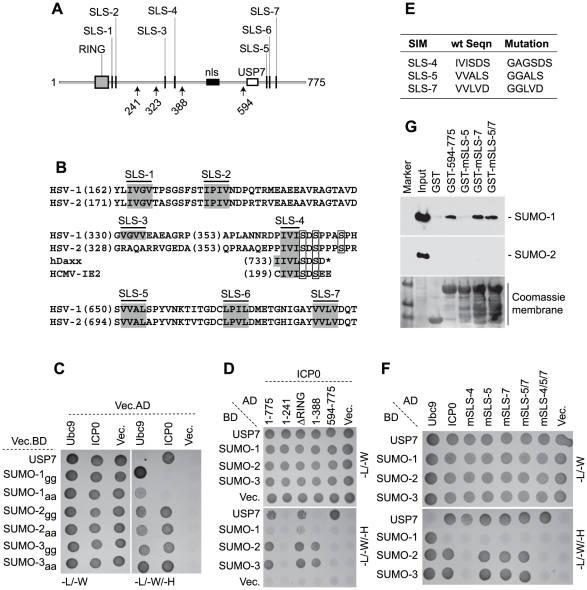
ICP0 interacts with SUMO in a SIM-dependent manner. (A) Locations of predicted SIM-Like Sequences (SLS, black vertical bars), the RING finger (grey box), nuclear localization sequence (nls, black horizontal bar), and USP7 binding domain (white box) within ICP0. Number arrows refer to amino acid coordinates. (B) Alignment of SLS in HSV-1 and HSV-2 ICP0. Grey boxes highlight the hydrophobic core within individual SLSs. SLS-4 is additionally aligned with SIMs of hDaxx and HCMV IE2. Square boxes adjacent to SLS-4 highlight phosphorylated serine residues. (C) Y2H analysis showing ICP0 interaction with SUMO-2/3, but not SUMO-1, in a C-terminal di-glycine independent manner. Ubc9 and USP7 are positive controls for SUMO and ICP0 interaction respectively. Mated diploids were plated out onto media lacking leucine and tryptophan (-L/-W, indicating the presence of both plasmids) or leucine, tryptophan, and histidine (-L/-W/-H). Positive interactions are indicated by growth on medium lacking histidine. *GAL4* activation domain (AD) or binding domain (BD) fusion orientations are highlighted. Vec indicates the respective empty vector control. (D) ICP0 interacts with SUMO-2/3 in a RING finger-independent manner and requires residues between amino acids 241 to 388. (E) Table highlighting residue mutations made within SLS-4, -5 and -7. (F) Mutation of SLS-4 inhibits ICP0’s ability to interact with SUMO-2/3 in Y2H assay. (G) GST pull down assay demonstrating that the C-terminal third of ICP0 (residues 594-775) interacts with SUMO-1.

As ICP0 displayed STUbL-like properties, we next analyzed its polypeptide sequence for the presence of SIMs and found seven potential SIM-like sequences (SLS), six that conform to the consensus (with the possible exceptions of SLS-2 and -6 that contain proline residues), and one (SLS-4) that has homology [IVISDS] to a SIM previously identified in hDaxx [38] and the HCMV regulatory protein IE2 [39] (Figures 3A and 3B). These predicted SLSs of ICP0 are distributed throughout the entire ORF, with SLS-1 and -2 close to the RING finger, SLS-3 and -4 in the middle and SLS-5 to -7 in the C-terminal third of the protein. SLS-4 lies adjacent to a known phospho-serine region (Figure 3B) that is required for ND10 disruption in certain cell types [40], [41]. Comparison of ICP0 sequences from HSV-1 and HSV-2 demonstrated that six of the predicted SIMs are conserved, the exception being SLS-3 (Figure 3B).

Consistent with the presence of potential SIMs, yeast two-hybrid (Y2H) assays demonstrated that ICP0 interacted with both SUMO-2 and -3 (Figure 3C). ICP0 did not interact with SUMO-1 in this assay system, even though SUMO-1 was capable of interacting with hDaxx in a SIM-dependent manner (Figure 3C and S5, [38]). USP7 was used as a positive control for ICP0 interaction [42]. The ICP0-SUMO interaction was not mediated through covalent linkage, as SUMO-2/-3 mutants lacking the C-terminal di-glycine motif still interacted with ICP0 (Figure 3C). Using a panel of C-terminal ICP0 deletion mutants we found that the SUMO-2/-3 interaction occurred in an ICP0 RING finger-independent manner and required residues 241-388 (Figure 3D). These data suggest that either SLS-3 or SLS-4 could mediate the SUMO-2/-3 interaction. As SLS-3 is not conserved in HSV-2 (Figure 3B), we investigated the requirement for SLS-4 and two other SIM-like sequences (SLS-5 and -7) for interaction with SUMO in the context of full-length ICP0. Mutation of SLS-4 abrogated the SUMO-2/-3 interaction in the Y2H system, but mutation of SLS-5 or -7, either individually or combined, had no effect (Figures 3E and 3F). We conclude that SLS-4 constitutes a genuine SIM that is specific for SUMO-2/3.

Given that the C-terminal third of ICP0 was shown to be required for the efficient degradation of SUMO-conjugates during infection (Figure S2C), *in vitro* pull-down assays were performed using purified C-terminal fragments of ICP0 (residues 594-775 linked to GST) in order to determine if these sequences mediated any interaction with SUMO (Figure 3G). In contrast to the Y2H analysis (Figure 3F), the C-terminal fragment of ICP0 interacted with SUMO-1, but not SUMO-2, in this assay. Individual mutation of SLS-5, but not SLS-7, disrupted this interaction. Surprisingly, the C-terminal region of ICP0 mutated in both SLS-5 and SLS-7 interacted with SUMO-1 at wt levels. We conclude from these data that the C-terminal portion of ICP0 is able to interact with SUMO-1 and that SLS-5 probably represents an authentic SIM for SUMO-1. However, further analysis will be required in order to define the basis of ICP0-SUMO-1 interaction within this region.

### ICP0 ubiquitinates SUMO-2 chains *in vitro* and induces the widespread degradation of SUMO-conjugates

**Figure 4:**
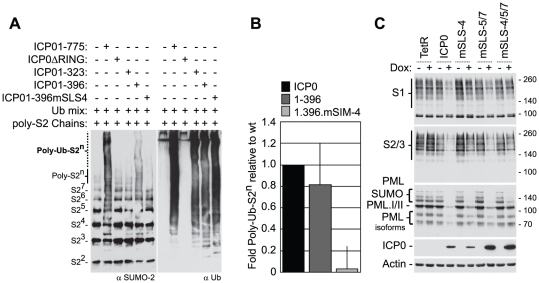
SLS-4 is required for the *in vitro* ubiquitination of poly-SUMO-2 chains and degradation of SUMO conjugates *in vivo*. (A) *In vitro* ubiquitination reactions were carried out in the presence of E1, UbcH5a and ubiquitin (Ubmix), poly-SUMO-2 chains and purified wt ICP0, ICP0ΔRING, or ICP0 C-terminal truncation mutants ICP01-323, ICP01-396 or ICP01-396mSLS-4 as indicated (-/+). Ubiquitinated products were analyzed by western blot for ubiquitinated SUMO species (left-) and poly-ubiquitin chain formation (right-hand panels, respectively). Poly-ubiquitinated SUMO-2 species are labelled Poly-Ub-S2^n^. Superscripts denote the number of SUMO-2 (S2) molecules within the chain. (B) Quantification of Poly-Ub-S2^n^ species (dotted line) detected by western blot analysis in reaction mixtures containing ICP0, ICP01-396, and ICP01-396mSLS-4 expressed as a relative fold decrease in relation to reaction mixtures containing wt ICP0. Error bars represent the standard deviation in Poly-Ub-S2^n^ levels detected over four independent experiments. (C) Analysis of SUMO conjugates and PML stability in cells induced to express wt ICP0, ICP0 mSLS-4, -5/7, -4/5/7 mutants, or empty vector control (TetR) cells at 24 hours post-induction with doxycycline (Dox; +) compared to uninduced controls (-). The actin and ICP0 blots provide loading and ICP0 expression controls.

The RING finger domain of ICP0 has E3 ubiquitin ligase activity *in vitro* in the presence of the E2 ubiquitin conjugating enzymes UbcH5a (UBE2D1) and UbcH6 (UBE2E1) [3], [43]. Ubiquitination reactions carried in the presence of purified poly-SUMO-2 chains demonstrated that ICP0 could catalyze the formation of high MW poly-SUMO-2 ubiquitin conjugates in a RING finger-dependent manner, which required sequences encompassing SLS-4 (compare ICP0.1-323 with ICP0.1-396, Figure 4A). Mutation of SLS-4 within the context of ICP0.1-396 significantly reduced ICP0’s ability to mediate the poly-ubiquitination of SUMO-2 chains *in vitro* (Figure 4A and B), even though this mutant had ubiquitin ligase activity equivalent to that of full-length ICP0 and ICP0.1-396 (Figure 4A, right-hand panels). Taken together, these data indicate that ICP0 can directly interact with and ubiquitinate poly-SUMO chains in solution and that SLS-4 plays a role in this process. It is of interest to note that while ICP0.1-396 can mediate the ubiquitination of poly-SUMO2 chains *in vitro*, this activity was reduced in comparison to full-length ICP0 (Figure 4A and B), suggesting that additional sequences and/or post-translational modifications may contribute to ICP0’s ability to ubiquitinate poly-SUMO2 chains in solution.

To investigate the role of the SLSs within ICP0 in the degradation of high MW SUMO-conjugates, inducible cell lines expressing wt or SLS mutant forms of ICP0 were analyzed for SUMO-conjugate abundance following doxycycline induction (Figure 4C). Expression of both wt and mSLS-5/7 ICP0 resulted in reduced levels of both SUMO-1 and SUMO-2/3 conjugates at 24 hours post-induction, whereas the mSLS-4 or mSLS-4/5/7 mutants did not. Analysis of PML stability demonstrated that wt, mSLS-4 and mSLS-5/7 forms of ICP0 could all induce the substantial degradation of PML, whereas this was much less marked in the case of the triple mutant ICP0-mSLS-4/5/7 (Figure 4C, an independent time course experiment is shown in Figure S3C). In agreement with infection data (Figure S3A), these results imply that ICP0 exhibits substrate selectivity with regard to SUMO-conjugated species, in that PML is targeted more efficiently than SUMO conjugates in general. Furthermore, since the defect of the triple mutant in PML degradation is substantially greater than either the single SLS-4 or the double SLS-5/7 mutations, it is possible that the SLSs are acting cooperatively. Mutation of SLS-5 and -7 in the C-terminal region of ICP0 greatly enhanced its accumulation after induction, and while the reasons for this remain unknown, it could result in an underestimation of any defects caused by these mutations. As an important control, we found that all SLS mutant forms of ICP0 were able to induce the formation of colocalizing conjugated ubiquitin (Figure S6), indicating that these mutations did not compromise the E3 ubiquitin ligase activity of ICP0 *per se*. Collectively, the data in Figures 3 and 4 indicate that ICP0 directly interacts with and ubiquitinates SUMO, and combined mutations within SLS-4, -5, and -7 reduce ICP0-dependent degradation of high MW SUMO conjugates. We conclude that ICP0 has STUbL-like properties.

### ICP0 induces the degradation of PML by both SUMO-targeted and SUMO-independent mechanisms

**Figure 5:**
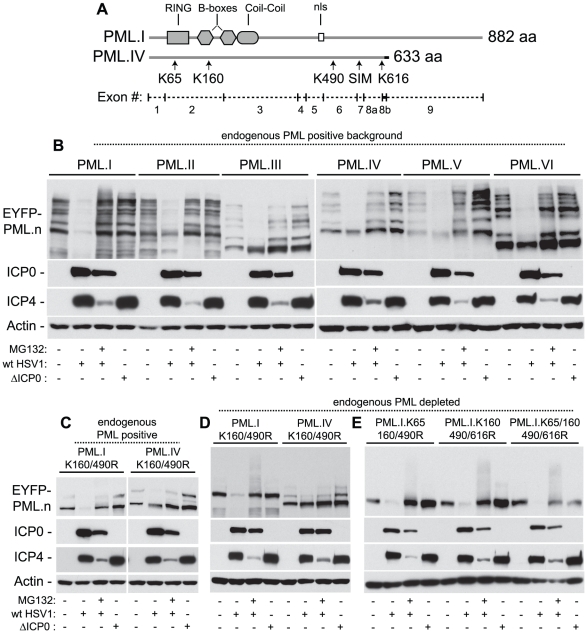
ICP0 preferentially induces the degradation of SUMO-conjugated PML. (A) Maps of PML.I and PML.IV depicting RING, B-box, coiled-coil and nls motifs. Arrows indicate the SIM and lysine (K) residues mutated in this study, vertical lines show exon boundaries in PML.I, and the black horizontal bar represents the PML.IV specific exon 8b. (B) ICP0 preferentially induces the proteasome-dependent degradation of SUMO-modified forms of PML.I-VI. Cells expressing EYFP-PML isoforms I-VI were infected with wt (MOI 2) or ICP0-null mutant HSV-1 (ΔICP0, MOI 10), in the presence or absence of MG132, and harvested at 6 hours post-infection. Cell extracts were analyzed by western blot for EYFP-PML in comparison with mock-infected controls. (C and D) ICP0 degrades EYFP-PML.I but not EYFP-PML.IV K160/490R mutants in both control cells (C) and cells depleted of endogenous PML (D). (E) ICP0 induces the degradation of EYFP-PML.I.K65/160/490/616R in a SUMO modification-independent manner.

We next tested whether ICP0 induces the degradation of PML in a SUMO modification-dependent manner. PML is expressed as a complex family of related isoforms that contain a SIM and multiple SUMO modification sites [24], [44], [45] (Figure 5A). Using a series of cell lines expressing individual EYFP-linked PML isoforms in normal or PML-depleted backgrounds [46], we found that the SUMO-modified forms of all PML isoforms were degraded in an ICP0- and proteasome-dependent manner during infection, consistent with previous studies analysing ICP0’s ability to induce the degradation of endogenous PML during infection [9]–[11]. In contrast, the unmodified forms of EYFP-PML were relatively resistant to degradation, with the exception of PML isoform I (Figure 5B). Individual EYFP-PML isoform degradation was not dependent on endogenous PML as similar results were obtained in PML-depleted cells (data not shown).

Using isoforms I and IV as example substrates, we investigated the requirement for SUMO modification for ICP0 induced degradation of PML. Lysine to arginine mutations at residues 160 and 490 (K160/490R), the two major SUMO modification sites [47], were expressed in control and PML-depleted cells and monitored for their respective stabilities during infection. In contrast to PML.IV.K160/490R, PML.I.K160/490R was readily degraded in an ICP0-dependent manner (Figures 5C and 5D). Although the double K160/490R mutation substantially reduced SUMO modification, some modified bands remained, particularly in the presence of endogenous PML. Inclusion of the K65R mutation (K65/160/490R), affecting the other published SUMO modification site [47], did not alter this banding pattern [17]. We noted that lysine 616 also falls within a good SUMO modification consensus sequence (LKID). Additional mutation of K616R (K160/490/616R), either in the presence or absence of the K65R mutation, reduced modification to undetectable levels in both endogenous and PML-depleted backgrounds (Figure 5E; [17]). This mutant form of PML.I remained equally sensitive to ICP0-mediated degradation (Figure 5E), indicating that sequences specific to exon 9 within PML.I (Figure 5A), either directly or indirectly, confer additional sensitivity to ICP0-mediated degradation independent of PML SUMO modification status. These data suggest that ICP0 utilizes dual targeting mechanisms to mediate the degradation of PML during infection, one being a SUMO-dependent mechanism that leads to the preferential degradation of all SUMO-modified PML isoforms, and the other a SUMO modification-independent mechanism that can target PML.I for degradation via sequences encoded by exon 9.

### SIM-like sequences within ICP0 contribute to its biological functions

**Figure 6:**
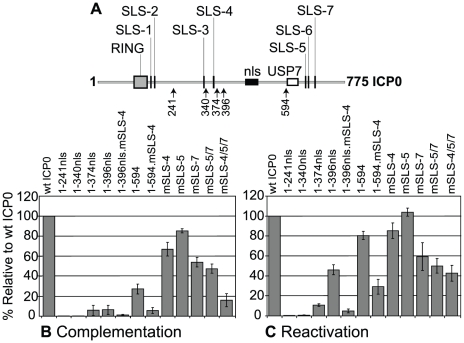
SLSs within ICP0 regulate its ability to complement and reactivate mutant HSV-1 viruses in cell culture. (A) As in Figure 3A. (B) Complementation of ICP0-null mutant HSV-1 plaque formation by prior induction of expression of various N-terminal fragments of ICP0 (as depicted in A) and full-length ICP0 carrying individual or combined SLS mutations in the inducible cell line system. The titre of a mutant virus stock was determined in each cell line and plotted with respect to that in cells expressing wt ICP0. Means and standard deviations of two to seven independent determinations are presented. (C) Analysis of ICP0 induced reactivation of gene expression from quiescent HSV-1 genomes. Cells were infected with multiply defective HSV-1 mutant *in*1374 to establish quiescently infected cultures, then 24 h later ICP0 expression was induced with doxycycline. Reactivation was assessed the following day by staining for β-galactosidase expression from the marker gene in the *in*1374 genome. The proportion of reactivated cells in each cell line was expressed as a percentage of that in cells expressing wt ICP0 following determination of positive cell numbers in three high magnification views of each sample. Means and standard deviations are presented.

We next tested the hypothesis that SLSs within ICP0 contribute to its ability to stimulate HSV-1 lytic infection and reactivation of gene expression from quiescence. Cell lines that express various mutants of ICP0 in an inducible manner were tested for their ability to stimulate plaque formation of a HSV-1 ICP0-null mutant virus and to reactivate gene expression from quiescent HSV-1 genomes. The use of these assays to analyze other mutants of ICP0 has been described in detail elsewhere [8]. N-terminal fragments 1–240nls or 1–340nls had negligible complementation activity in ICP0-null mutant virus plaque assays, whereas fragments 1–374nls, 1–396nls and 1–594, that include SLS-4, exhibited detectible levels of complementation activity (Figure 6B). Mutation of SLS-4 within constructs 1–396nls and 1–594 virtually eliminated complementation, indicating that SLS-4 contributes to ICP0 activity in the context of these shorter fragments. Individual mutation of SLS -4, -5 or -7 in full-length ICP0 had varying but lesser effects on ICP0 complementation efficiency. However, similar to PML degradation (Figure 4C and S3C), the triple mutant was significantly less active than wt ICP0 (Figure 6B).

In assays monitoring the reactivation/derepression of β-galactosidase gene expression from cells harbouring quiescent HSV-1 genomes, large reductions in activity resulted from mutation of SLS-4 in the 1-396nls and 1–594 backgrounds, again highlighting the importance of SLS-4 in the context of these shorter ICP0 fragments (Figure 6C). Comparison of the effects of individual and combined SLS mutants in the full-length ICP0 again demonstrated varying levels of activity, with the triple SLS mutant being the most defective with over a 50% drop in reactivation efficiency (Figure 6C). We have observed that mutant ICP0 proteins frequently retain greater relative activity in this reactivation assay than in the complementation assay [8], [48]. We conclude that SIM-like sequences within ICP0 significantly contribute to its biological functions, both in its ability to complement ICP0-null mutant plaque formation and, to a lesser extent, the reactivation of quiescent HSV-1 gene expression. However, it is likely that additional sequences within the C-terminal third of ICP0 also contribute to its functionality in these assay systems.

### ICP0 and its viral orthologues induce the proteasome-dependent degradation of SUMO conjugates independent of infection

We have shown that related RING finger viral orthologues of ICP0, including BICP0 (from bovine herpes virus type 1), EICP0 (from equine herpes virus type 1), and PICP0 (from pseudorabies virus), have E3 ubiquitin ligase activity *in vitro* and can partially substitute the functional requirement for ICP0 during HSV-1 ICP0-null mutant infection [48]. Importantly, these viral orthologues were shown not only to inhibit the recruitment of PML to sites associated with incoming HSV-1 viral genomes, but also to modulate the SUMO conjugation profile of PML and/or Sp100 [48]. We therefore investigated whether STUbL-like properties were a conserved activity of the ICP0 family of RING finger proteins.

**Figure 7:**
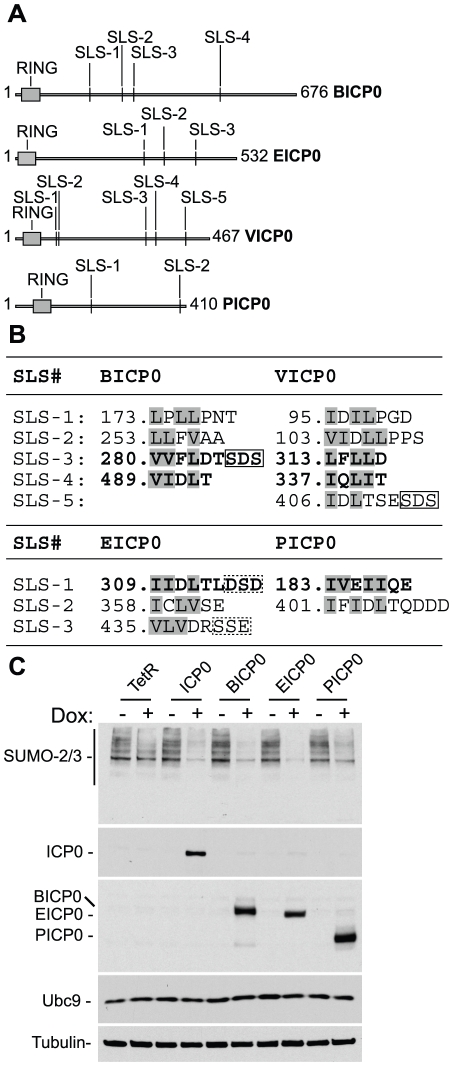
ICP0 and its viral orthologues induce the degradation of SUMO conjugates independently of virus infection and the degradation of Ubc9. (A) Schematic representation of ICP0 orthologue proteins BICP0, EICP0, VICP0 and PICP0 illustrating the distribution of putative SIM-Like Sequence (SLS) motifs in relation to their respective RING finger domains (grey boxes). Numbering reflects the first and last amino acid within each ORF. (B) Amino acid sequence of putative SLS motifs identified within each ICP0 viral orthologue. Numbers refer to the coordinates of the first amino acid shown within each SLS with respect to their individual ORF sequence. Bold lettering represents motifs that conform to the SIM consensus as described [24]–[26]. Boxed sequences show conserved triplets composed of serine or acidic residues. (C) Cell lines induced to express ICP0, myc-tagged viral orthologues (BICP0, EICP0 and PICP0), or control cells (TetR) were analyzed for Ubc9 expression levels in relation to SUMO conjugate abundance 24 hours after treatment with doxycycline (0.1 µg/ml) (−/+). Blots were reprobed for ICP0, myc-tagged orthologue expression, and tubulin as a loading control.

All ICP0 viral orthologues, including VICP0 from varicella zoster virus, contain multiple putative SIM-like sequences, with at least one SLS conforming to the SIM consensus (Figure 7A and 7B bold highlights, [49]). SLS-3 of BICP0 also shares homology (Figure 7B, solid box) with the phospho-serine region downstream of SLS-4 of ICP0 (IVISDS, Figure 3B). We note that the other viral orthologues also contain similar acidic and/or serine amino acid sequences following some of their putative SIM-like sequences (Figure 7B, dashed boxes). These negatively charged motifs are similar to other examples that have been shown to enhance SIM-SUMO interactions [27], [28], [50].

Utilizing inducible cell lines that express ICP0 or related viral proteins, we found that like ICP0 (Figure 4C), BICP0, EICP0, and PICP0 were all capable of reducing the overall abundance of high MW SUMO conjugates following doxycycline induction (Figure 7C and S7). As found previously [48], VICP0 was expressed at insufficient levels to be active in this experimental system (Figure S7). Ubc9 levels remained unaltered in cells expressing ICP0 or related viral orthologues (Figure 7C), indicating that the degradation of SUMO conjugates could not be explained by indirect degradation of Ubc9. We conclude that the induced loss of SUMO conjugates by ICP0 and its viral orthologues occurs independently of viral infection and that STUbL-like activity is a conserved property of these related viral RING finger ubiquitin ligases.

## Discussion

In this report we demonstrate that the viral ubiquitin ligase ICP0 has STUbL-like properties that contribute to its ability to counteract host-cell intrinsic resistance to HSV-1 infection. Intrinsic resistance represents the first line of intracellular antiviral defence and, unlike innate or acquired antiviral immunity, is mediated by pre-existing cellular factors that attempt to restrict viral replication during the initial stages of infection (for reviews see [14], [51], [52]). In the case of herpesviruses, intrinsic resistance leads to the repression of viral gene expression, which may reflect an important biological aspect of how these viruses attain a quiescent state of infection prior to the establishment of latency. Whilst the role of chromatin modification on viral gene transcription has been investigated in many laboratories, we have identified an additional aspect to intrinsic resistance (not necessarily unconnected) that involves the SUMO conjugation pathway and components of ND10 [12], [13], [15], [16], [31].

**Figure 8:**
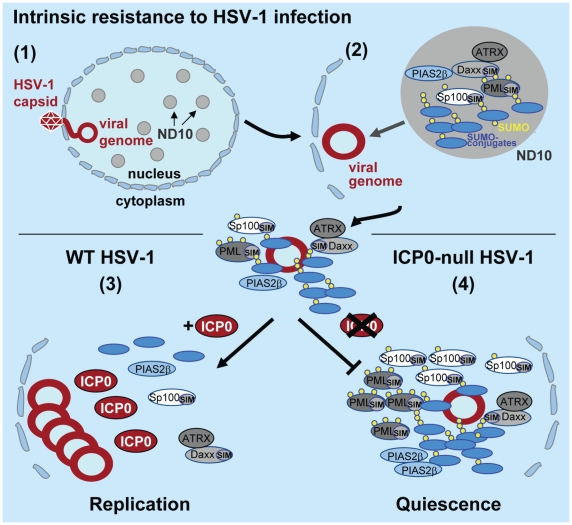
Model depicting the regulation of intrinsic antiviral resistance to HSV-1 infection mediated by the SUMO conjugation pathway. (1) During the initial stages of HSV-1 infection viral genomes enter the nucleus of infected cells. (2) Major ND10 components including PML, Sp100, hDaxx, and ATRX are recruited into foci that are closely associated with incoming HSV-1 genomes. This recruitment is dependent upon the SUMO conjugation pathway (Figures 1 and S1) and SIMs within PML, Sp100 and hDaxx [17]. (3) During wt HSV-1 infection, the STUbL-like activity of ICP0 promotes the preferential degradation of SUMO-conjugated proteins leading to the dispersal of restriction factors and the efficient onset of viral replication. (4) In the absence of ICP0, specific SUMO-conjugated proteins mediate the transcriptional repression of viral gene expression leading to the establishment of viral quiescence and latency.

We have recently shown that recruitment of PML and hDaxx to foci associated with incoming viral genomes is dependent upon their SIMs, and that additional SUMO-2/3 conjugates, as well as the SUMO E3 ligase PIAS2_β_, are also recruited to viral genomes in a PML-independent manner [17]. Consistent with these observations, we show here that depletion of Ubc9, the sole SUMO E2 conjugating enzyme, restricts the cell’s ability to repress ICP0-null mutant virus replication (Figure 1C and 1D) and inhibits the recruitment of SUMO conjugates and PML to foci associated with incoming viral genomes (Figure S1). Collectively these data demonstrate that the SUMO conjugation pathway plays a role in intrinsic resistance to HSV-1 infection. This phenotype is consistent with previously documented roles for SUMO conjugation in transcriptional repression (reviewed in [53]), the assembly of ND10 [38], [44], and the recruitment of chromatin modifying enzymes into ND10 [54]. It is therefore likely that in the context of intrinsic antiviral defence this pathway is involved in the assembly of a network of repressive factors that associate with viral genomes following their entry into the cell nucleus in order to bring about repression of viral transcription (summarized in Figure 8).

Analogous to viral counter measures against innate and acquired immunity, viruses have evolved mechanisms to disarm intrinsic antiviral defence. One of the first proteins to be expressed during HSV-1 infection is ICP0, a viral RING finger ubiquitin ligase that localizes to and disrupts ND10 by mediating the degradation of PML, its SUMO-modified isoforms, and SUMO-modified Sp100 [9]–[11]. The degradation and dispersal of ND10 constituent proteins correlates well with ICP0’s ability to counteract intrinsic defence, thereby aiding the efficient initiation of viral replication. However, the precise mechanism(s) by which ICP0 targets these ND10 proteins for degradation has remained elusive. Given the parallels between ICP0 and the cellular STUbL RNF4 [22], [23], [29], we decided to investigate if ICP0 possessed STUbL-like properties that contribute to its ability to counteract intrinsic defence. Whilst we show that ICP0 shares some phenotypic similarities to RNF4, a number of important differences have also been highlighted. Like RNF4, ICP0 preferentially induces the degradation of SUMO-modified forms of PML, but unlike RNF4 [23], [30], ICP0 is able to target PML without the need for additional PML SUMO modification (Figures 5B and S3A). In addition, ICP0 also induces the widespread degradation of SUMO-1 and SUMO-2/3 conjugate proteins during infection (Figure 2A and S2B). Importantly, this activity is not dependent upon the presence of PML (Figure S3B), indicating that this phenotype is not an indirect consequence of ND10 disruption. These data demonstrate that ICP0 targets additional SUMO-modified proteins for degradation other than those constitutively modified at ND10, consistent with its ability to localize to SUMO-2/3 conjugates in PML-depleted cells during the initial stages of infection (Figure 2E). These differences, plus others described below, indicate that ICP0 does not represent a precise viral orthologue of RNF4.

Although ICP0 contains many SIM-like sequences, only one of these was strongly identified as a functional SIM (SLS-4, IVISDS). This motif shares homology to previously characterized SIMs within hDaxx and the HCMV IE2 protein (Figure 3B; [38], [39]). SLS-4 is required for ICP0’s ability to interact with SUMO-2 (Figure 3F), ubiquitinate poly-SUMO-2 chains *in vitro* (Figure 4A), and to reduce the level of SUMO conjugates when expressed by itself in an inducible cell line system (Figure 4C). These data support the hypothesis that ICP0 has STUbL-like activity. It is of interest to note that serine residues adjacent to SLS-4 can be phosphorylated [41] and are required for ICP0’s ability to disperse PML in transfection based assays in certain cell types [40]. It is plausible therefore that this particular SIM is regulated by phosphorylation, similar to those previously identified in SUMO ligases [27]. Whilst the extent to which the other SIM-like sequences contribute to SUMO interaction remains unclear, our data indicate that the C-terminal third of ICP0 can additionally interact with SUMO-1, potentially in a SLS-5 dependent manner (Figure 3G). However, we note that this region influences many aspects of ICP0 function, including USP7 and CoREST binding [42], [55], ICP0 multimerization and ND10 localization [56].

Whilst ICP0’s STUbL-like activity is important for its biological functions (Figures 6B and 6C), it is important to note that ICP0 also directly interacts with and ubiquitinates other cellular proteins such as RNF8, USP7 and p53 in a SUMO modification-independent manner [43], [57], [58]. Here we found that PML.I can also be targeted for degradation in a SUMO modification-independent manner (Figures 5B–E). Thus, ICP0 clearly has both SUMO-dependent and -independent targeting specificities that may have an overall accumulative effect on counteracting intrinsic antiviral resistance. Furthermore, since SUMO-modified PML is degraded more rapidly than the bulk of SUMO conjugates, it appears that the identity of a SUMO-modified protein also influences ICP0 substrate targeting. Indeed, it is possible that optimal ICP0 targeting involves both SUMO- and substrate-dependent interactions that synergize to define the most avid ICP0 targets. Another important consideration is the distinction between biochemical targeting and spatial localization. SUMO-modified PML may be degraded more rapidly than other SUMO conjugates because ICP0 interacts more avidly with the former than the latter, or because the high concentration of ICP0 within ND10 preferentially enhances degradation of SUMO conjugates within these structures. While these two factors may well be related, we note that ICP0 colocalizes with SUMO-2/3 conjugates even in the absence of PML (Figure 2E). Similarly, the reduced activity of the C-terminal deletion mutant ICP0.1-594 on both PML [9] and SUMO conjugates (Figure S2C) could be due to less efficient biochemical targeting at a molecular level (Figure 3G), or because this mutant is diffusely distributed in the nucleus and not spatially targeted to ND10 where intra-nuclear SUMO conjugates accumulate.

Viruses have evolved numerous mechanisms to exploit ubiquitin conjugation in order to create cellular environments that favour viral replication. Our data identify a dual targeting (SUMO- and substrate-dependent) mechanism through which ICP0 manipulates the cellular environment in favour of HSV-1 replication. Given that the STUbL-like properties of ICP0 appear to be a conserved activity of this family of viral RING finger ubiquitin ligases (Figure 7C), it would be interesting to determine if this is a common mechanism for substrate targeting utilized by other viral ubiquitin ligases. The observation that ICP0 targets SUMO-conjugated proteins in general for proteasome-mediated degradation provides a plausible explanation for its ability to inhibit multiple factors involved in intrinsic antiviral defence (summarized in Figure 8). This activity may also account for other proposed roles for ICP0 in regulating other cellular pathways, including innate interferon-mediated defence and DNA damage response pathways, as components of these pathways have been shown to be regulated by SUMO modification [59], [60], [61], [62]. We stress, however, that the target specificity of ICP0 is regulated by factors in addition to the presence of conjugated SUMO, and that the biologically relevant targets will comprise only a minority of total SUMO-conjugated proteins. Nonetheless, our observations suggest a more general role for SUMO conjugation in resistance to pathogen infection. Evidence in support of this hypothesis includes: (i) that the chicken adenovirus protein Gam-1 inactivates the SUMO conjugation pathway by targeting the SUMO E1 activating enzyme complex in order to stimulate viral transcription [63]; (ii) that a pathogenic bacterium impairs the SUMO modification pathway to enhance infection [64]; and (iii) that SUMO modification of transcriptional regulatory proteins is frequently associated with transcriptional repression [53]. Since the recruitment of ND10 components to sites associated with viral genomes occurs extremely quickly and is independent of viral transcription [16], we propose that the SUMO pathway may regulate a process that responds to the entry of foreign DNA in general into the cell nucleus. Further investigation into ICP0’s SUMO-targeted and SUMO-independent ubiquitin ligase activities will provide insight into the cellular processes that regulate this response to infectious pathogens.

## Materials and Methods

### Cells

Human foetal foreskin diploid fibroblasts (HFs) were grown in Dulbeccos Modified Eagles Medium with 10% fetal calf serum (FCS). HepaRG cells [65] were grown in Williams Medium E with 10% fetal bovine serum Gold (PAA Laboratories Ltd), 2 mM glutamine, 5 µg/ml insulin and 0.5 µM hydrocortisone. All cell growth media contained 100 units/ml penicillin and 100 µg/ml streptomycin. PML-depleted and control HepaRG and HFs cells were described previously [12], [13]. Control and PML-depleted HepaRG cells reconstituted with individual PML isoforms expressed at close to endogenous levels and derivatives expressing PML isoforms I and IV with lysine to arginine substitutions at the known SUMO modification sites and at lysine residue 616 have been described previously [46]. PML isoforms are named according to [45]. Tetracycline inducible HepRG cells expressing ICP0 or alpha herpes viral orthologues have also been described previously [48]. Ubc9-depleted cells were constructed by lentiviral transduction, as described in [13] expressing a shRNA based upon a 19-mer (5’ GAAGTTTGCGCCCTCATAA 3’) within the Ubc9 open reading frame.

### HSV-1 strains and plaque assays

Wild type HSV-1 strain 17syn+, its ICP0-null mutant derivative *dl*1403 [66], RING finger deletion mutant FXE [67], and C-terminal truncation mutant E52 (expressing ICP0.1-594 [56]) were grown and titrated as previously described [68]. Derivatives of wt (*in*1863) and ICP0-null mutant (*dl*1403/CMV*lac*Z) HSV-1 that contain a β-galactosidase gene linked to the human cytomegalovirus immediate-early promoter/enhancer inserted into the *tk* locus were used for plaque assays as described [12].

### Western blot analysis and antibodies

Cells in 24-well dishes at 1×10^5^ cells per well were washed with phosphate buffered saline before harvesting in SDS-PAGE loading buffer. Proteins were resolved by SDS-PAGE and transferred to nitrocellulose membranes for western blotting. Monoclonal antibodies utilized recognised the following proteins: actin (AC-40, Sigma-Aldrich), tubulin (T4026, Sigma-Aldrich), ubiquitin (P4D1, Santa Cruz), ICP0 (11060, [69], UL42 [70], ICP4 (58S, [71], myc (9E10 sc-40, Santa Cruz) and PML 5E10 [72]. Rabbit polyclonal antibodies were used to detect Sp100 (SpGH [73]), PML (sc-9863, Santa Cruz), Ubc9 (ab30505, AbCam); EGFP (ab290, Abcam), SUMO-1 (ab32058, Abcam), and SUMO-2/3 (ab3742, Abcam).

### Immunofluorescence and confocal microscopy

Cells on 13 mm glass coverslips were fixed and permeabilized using 2.5% non-buffered formaldehyde and 0.5% Triton-X100 in 10 mM HEPES (pH 7.0), 100 mM NaCl, 300 mM sucrose, 3 mM MgCl_2_, 5 mM EGTA. The secondary antibodies used were Alexa 488, 594, and 633 conjugated donkey anti-rabbit, - sheep, and -mouse IgG (Invitrogen). A glycerol-based mounting medium was used (Citifluor AF1). The samples were examined using a Zeiss LSM 510 confocal microscope with 488 nm, 543 nm and 633 nm laser lines and a x63 Plan-Apochromat oil immersion lens, NA 1.40. Exported images were processed using Adobe Photoshop with minimal adjustment and assembled for presentation using Adobe Illustrator.

### Yeast-two hybrid (Y2H) analysis

Y2H analysis was based upon the Matchmaker 3 system (Clontech) using AH109 and Y187 yeast strains. ICP0 cDNAs encoding wt ICP0.1–775, ICP0.1–775ΔRING (FXE), ICP0.1–388 and 1–241, along with hDaxx and hDaxx.mSIM (C-terminal SIM mutant aa 733–740 IIVLSDSD to IGAGSDSD, [17]) were cloned into pGAD-T7 in frame with the *GAL4* activation domain (AD). cDNAs encoding SUMO isoforms, their inactive conjugation mutants (-GG to -AA), pp71 and USP7 were cloned into pGBK-T7 in frame with the *GAL4* DNA binding domain (BD). Transformed yeast colonies were picked, mated overnight, and diploids serially diluted prior to plating out onto selective medium (as highlighted) following the manufacturer’s guidelines. Colonies were allowed to grow for 72 hours prior to image capture.

### Protein purification and *in vitro* assays

Full-length poly-histidine tagged ICP0 and ICP0ΔRING were purified as previously described [3]. Poly-histidine tagged ICP0.1–323, ICP0.1–396, ICP0.1–396SLS-4, SUMO-1 and SUMO-2 were purified from bacterial extracts utilizing Nickel agarose affinity chromatography and dialysed into 50 mM Tris (pH 7.5), 150 mM NaCl, 2.5% glycerol, 2 mM MgCl_2_, 1 mM DTT. Poly-HisSUMO-2 chains were purchased from Boston Biochem. *In vitro* ubiquitination reactions were carried out in the presence of 20 ng poly-HisSUMO-2 chains as essentially described in [43] utilizing 10 ng E1 activating enzyme, 30 ng UbcH5a, 2.5 µg ubiquitin (Sigma-Aldrich), and 100 ng ICP0, ICP0ΔRING, ICP0.1–323, or ICP0.1.396 in the presence of 50 mM Tris (pH 7.5), 50 mM NaCl, 1 mM MgCl_2_ and 5 mM ATP. Reactions were carried out at 37°C for 90 minutes and terminated by the addition of boiling mix containing 8 M urea and 100 mM DTT. Quantification of poly-ubiquitinated poly-SUMO2 chains was performed by densitometry analysis of western blots using Quantity One software (Bio-Rad).

### Interaction analysis

Glutathione-S-Transferase (GST) pull-downs were carried out in buffer H (50 mM HEPES pH 7.0, 150 mM NaCl, 5 mM β-mercaptoethanol and 0.1% NP-40) using beads bound to either GST alone or GST linked to the C-terminal 594-775 amino acids of ICP0 (GST-E52; [56]) or equivalent fragments with mutations in SLS-5, -7, or -5/7 in 1.5 ml of precleared bacterial supernatants containing either His-tagged SUMO-1 or SUMO-2 for 90 minutes end-over-end at 4°C. The beads were washed three times in 1 ml buffer H and soluble complexes were eluted in 60 µl of 1x SDS-PAGE loading buffer.

### ICP0 inducible cells lines

HepaRG cells expressing wt and 1–594 C-terminal truncation mutant ICP0 proteins in an inducible manner have been described previously [8]. A series of C-terminal truncation mutants of ICP0 were constructed using existing restriction sites or previously described EcoRI linker insertion mutants [5] as follows: ICP0.396nls and ICP0.1–517 used the NotI or MluI sites in the ICP0 cDNA; ICP0.1–547, 1–374nls and 1–340nls used linker insertions E1, E51 and E15; ICP0.1–241nls used the ICP0 truncation fragment including the first 241 codons described previously [3]. ICP0.1–547 and ICP0.1–517 include the normal ICP0 nuclear localization signal and the constructs include a C-terminal linker containing stop codons. The shorter truncation mutants (with nls in the name) contain a C-terminal linker encoding the SV40 T-antigen nuclear localization signal followed by a stop codon. Substitution mutants in SLS-5 (VVAL to GGAL) and SLS-7 (VVLV to GGLV) were constructed by a PCR splicing approach. MluI-SalI fragments containing the desired mutations were transferred into the wt ICP0 lentivirus expression vector. Substitution mutants in SLS-4 (PIVI to PGAG) were constructed by oligonucleotide synthesis of the region between the SfiI and NotI sites in the ICP0 cDNA, maintaining the coding potential except for the desired mutations, while decreasing the GC content by introduction of silent mutations, then rebuilding the oligonucleotides into the wt cDNA. The double mutant mSLS-5/7 was prepared by serial mutagenesis. The mSLS-4 mutants in the 1-594 and 1-396nls truncations and the mSLS-5/7 double mutant were constructed using the NotI site on the 3’ side of the mutated SLS-4 motif. All mutants were confirmed by extensive DNA sequence analysis after insertion into lentiviral vectors. Cell lines expressing these proteins were isolated as described previously [8]. Assays of complementation of plaque formation by ICP0-null mutant HSV-1 and derepression of quiescent HSV-1 after induction of ICP0 expression were performed and quantified as described [8].

## Supporting Information

Figure S1 Ubc9 expression is required for the efficient recruitment of SUMO and PML to sites associated with incoming HSV-1 genomes in the absence of ICP0. Control (shNeg) or Ubc9-depleted (shUbc9) HFs were infected with an ICP0-null mutant virus (ΔICP0) and the recruitment of SUMO-1, SUMO-2/3, and PML proteins (green) to sites associated with incoming viral genomes (as shown by ICP4 staining, red) was analyzed in cells at the periphery of developing plaques. Scale bar represents 5 µm.
(EPS)

Figure S2 The C-terminal third of ICP0 is required for the efficient degradation of high MW SUMO conjugates during HSV-1 infection. (A) As in Figure 3A. (B) Western blots of HFs infected with wt or ICP0-null (ΔICP0) mutant HSV-1 at MOI of 5 pfu per cell in the absence or presence (−/+) of MG132 and harvested at the indicated times post-infection (Hrs PI). Other details are as described in Figure 2. (C) Analysis of SUMO conjugate abundance in HFs infected with wt or C-terminal ICP0 truncation mutant (ICP0.1–594) infected at various MOI at 8 hours PI.
(EPS)

Figure S3 ICP0 induces the proteasome-dependent degradation of SUMO conjugates in a PML-independent manner. (A) HFs and HepaRG cells were infected with wt HSV-1 at an MOI of 2 pfu per cell and harvested at the indicated times post-infection (Hrs PI). The abundance of SUMO conjugates and PML was analyzed. ICP0 and actin provide the infection and loading controls, respectively. (B) Control (shLuci) or PML-depleted (shPML) HFs were infected with wt HSV-1 or ICP0-null (ΔICP0) mutant virus at an MOI of 5 pfu per cell in the absence or presence (−/+) of MG132. Cells were harvested at the indicated times post-infection (Hrs PI) and analyzed for SUMO-1 and SUMO-2/3 conjugate abundance. Blots were reprobed for viral antigens ICP0, ICP4 and UL42, and tubulin as a loading control. (C) Time course analyzing PML stability in cells induced to express wt ICP0 or ICP0 mSLS-4, −5/7, −4/5/7 mutants at various time points post-induction (hours) with doxycycline (0.1 µg/ml Dox). The actin and ICP0 blots provide loading and ICP0 expression controls.
(EPS)

Figure S4 ICP0 localizes to SUMO-2/3 conjugates in a PML-independent manner during the early stages of HSV-1 infection. (A–D) The separated channels of the images from Figures 2B-E, including the channel for PML as a control for PML depletion, showing the relative localization of ICP0 (green), SUMO conjugates (red), and PML (cyan) in control (shLuci) and PML-depleted (shPML) cells at the periphery of developing plaques at early times post-infection with wt HSV-1. Nuclei were stained with DAPI. The insert at the lower right corner shows an expanded region highlighted by the white box.
(EPS)

Figure S5 SUMO-1 interactions with hDaxx, but not ICP0, in a SIM-SUMO dependent manner in the yeast-two-hybrid system. Mated diploids expression fusion proteins of interest (as highlighted) were plated on to selective media (as described in Figure 3C) either in the presence or absence of 1 mM 3-aminotriazole (3-AT). *GAL4* activation domain (AD) or binding domain (BD) fusion domain orientations are indicated. Vec indicates the empty vector control. USP7 and pp71, known binding partners of ICP0 and hDaxx respectively [42], [74], were used as positive controls for interaction. Wt hDaxx and hDaxx.mSIM, a C-terminal SIM mutant (aa 733–740 IIVLSDSD to IGAGSDSD) was used as a control for SIM-SUMO-1 interaction.
(EPS)

Figure S6 ICP0 SLS mutants induce the colocalization of conjugated ubiquitin within cell nuclei. Cells were induced to express wt ICP0, ICP0 mSLS-4, -5/7, or -4/5/7 (green) and analyzed for their respective abilities to colocalize with conjugated ubiquitin (conj. Ub, red) as detected by FK2 staining by confocal microscopy. Scale represents 5 µm.
(EPS)

Figure S7 ICP0 and its viral orthologues induce the degradation of both SUMO-1 and SUMO-2 conjugates independently of virus infection. Cell lines induced to express ICP0, myc-tagged viral orthologues (BICP0, EICP0, PICP0 and VICP0), or control cells (TetR) were analyzed for SUMO-1 and SUMO-2/-3 conjugate abundance 24 hours after treatment with doxycycline (0.1 µg/ml; −/+). Blots were reprobed for ICP0, myc-tagged orthologue expression, and actin as a loading control.
(EPS)
